# Transactions of the Chicago Society of Physicians and Surgeons

**Published:** 1873-12-01

**Authors:** 


### Society Reports.

### TRANSACTIONS OF THE CHICAGO SOCIETY OF PHYSICIANS AND SURGEONS.

MEETING OF NOVEMBER 10, 1873.

*Reported by Plym. S. Hayes, M.D.*

This Society was organized after the fire for the benefit of physicians resident in the southern portion of the city; but has since added to its numbers those practicing in both the North and West Divisions, and increased to such an extent that it was considered desirable to hold its meetings in a more central location. For this reason the meeting was called in the Grand Pacific Hotel.

The meeting was called to order at 8 P.M., by the Vice-President, in the absence of the President, Dr. A. Fisher. There were forty present, including the President, Secretary, and several members of the Chicago Medical Society, and some representatives of the Illinois State Microscopical Society.

The Chairman of the Committee on Permanent Place of Meeting reported on the subject in full. The report was accepted, the Committee discharged, and the final consideration of the subject deferred to the next regular meeting.

Dr. John Bartlett then read a paper, entitled, "On a Marsh Plant from the Mississippi Ague-Bottoms, supposed to be kindred to the Gemiasma of Salisbury, with a Consideration of its Genetic Relations to Malarial Diseases."

The first section of the paper reviewed Salisbury's article on the Palmella Plant, which appeared in 1866 in the *American Journal of the Medical Sciences*, and concluded with an account of Dr. Bartlett's observations at Keokuk in 1871, and at Riverside and Lemont during the summer of 1873, when no fungi were discovered, and no malarial fevers prevailed in the two places named.

The second section set forth the relation of the plants to the endemics of East Keokuk. In 1871 the Mississippi bottoms were covered with an abundant growth of these fungi, and ague was exceedingly prevalent. During the early portion of the last season the plants were very scarce, and could be found only after a prolonged search. At this time there was little malarial fever. Later in the season the "ague-plants" were more abundant than in 1871, and miasmatic disorders were as frequent and severe as during the preceding endemic.

The third section gave a description of the plant. Its appearance, in what seems to be the first stage of its growth, corresponded to the hygrogastrum of Rabenhorst. It had, however, other phases of development than those described by this botanist. The globular portion of the plant collapsed, after which the cells composing the globe-wall developed and extruded spores. From these spores were developed two remarkable features, viz.: crystalloid bodies and crystalline, mycelium-like threads. From these crystalline developments sprang the mother-plant, thus completing the cycle of growth. By far the most interesting feature of this plant, however, was the character of its spore-contents. These spores were not seed. They contained a protoplasmic fluid, in which appeared germinal particles, minute cells, rapidly multiplying and forming the crystalloid bodies and crystalline threads. The spores, as well as the free germinal particles, were caught on slides placed over the plant at night. The germinal atom was exceedingly small—less than one fifteen-thousandth part of an inch. These atoms were detected by Dr. Bartlett in his own blood and secretions, as well as in the blood and excretion of ague patients. In blood and urine left upon a slide the multiplication of the atoms was rapid, as was also the development (from the particles) of the crystalloid substances above described.

The fourth section considered the zymotic character of malarial fevers, and adduced the facts going to show that the exciting cause was the "germinal atoms" of the fungi. The history of malarial fevers was reviewed, and an explanation given of the organic lesions which they occasion. He thought that the "germinal atoms" operated upon the blood in the manner of a ferment, appropriating nutritious material for their use and multiplication, as the yeast and vinegar-plants consume the fluid in which they grow.

A fifth section referred to the light thrown by this discovery upon the etiology of cholera and yellow fever.

The sixth and last section considered the prophylactic treatment of ague, so far as regarded destroying or rendering innocuous the malarial fungi. This was to be effected by flooding them with water (which destroyed their virulent power); by covering them with ashes or similar substances; by surrounding them with foliage, particularly that of the pine and eucalyptus globulus; and finally, by the use of some remedy yet to be discovered, which would destroy the germinal atoms after their introduction into the system, without danger or inconvenience resulting from its continued use.

Dr. Bartlett concluded by a reference to the discoveries of Dr. Salisbury. He was inclined to regard the palmellæ, imperfectly described by Prof. Salisbury, as the same cells which may be seen in one phase of the growth of the plant here referred to. He regarded the minute cell described by Salisbury as the "spore" of the palmellæ, as nothing else than the germinal particle discovered by himself: though Salisbury described it as having a nucleus and cell-wall, while neither of these elements of structure were apparent to Dr. Bartlett.

On the conclusion of the reading, the Society adjourned, and an opportunity was afforded for the inspection of the microscopical preparations of the plants in every stage of development; of the "germinal atoms" visible in the blood of the essayist, who had for some weeks past experienced symptoms of remittent fever, in consequence of the exposure incidental to his extended researches; and colored sketches exhibiting the plant as it appears when growing in the mud of the ague-bottoms.

---

MEETING OF NOVEMBER 24TH, 1873.

The Society met in Parlor No. 1 of the Grand Pacific Hotel, the Vice-President in the chair. The minutes of the preceding meeting were read and approved.

The determination of the question as to the *permanent* place of meeting being the special order of the hour, Dr. Powell moved that that portion of the Committee's report having refence to the proposal of the proprietors of the Grand Pacific Hotel be accepted, and that hereafter the Society shall meet in the central portion of the city, and in the Grand Pacific Hotel. The motion prevailed with a large majority.

Five names of physicians were proposed for membership.

Dr. Hyde then read a review of John Hall's translation of Lanfranc's Surgery, published in London in 1565, in black letter. The volume had been recently procured of an antiquary in England by Dr. Chas. Gilman Smith, of this city. Various extracts were read from the pages of the work in connection with an historical sketch of the condition of surgery and anatomical science in the middle of the 16th century.

The Socity then adjourned.

